# Insulin-Like Growth Factor-I E-Peptide Activity Is Dependent on the IGF-I Receptor

**DOI:** 10.1371/journal.pone.0045588

**Published:** 2012-09-21

**Authors:** Becky K. Brisson, Elisabeth R. Barton

**Affiliations:** Department of Anatomy and Cell Biology, School of Dental Medicine, University of Pennsylvania, and Pennsylvania Muscle Institute, University of Pennsylvania, Philadelphia, Pennsylvania, United States of America; Aix-Marseille University, France

## Abstract

Insulin-like growth factor-I (IGF-I) is an essential growth factor that regulates the processes necessary for cell proliferation, differentiation, and survival. The *Igf1* gene encodes mature IGF-I and a carboxy-terminal extension called the E-peptide. In rodents, alternative splicing and post-translational processing produce two E-peptides (EA and EB). EB has been studied extensively and has been reported to promote cell proliferation and migration independently of IGF-I and its receptor (IGF-IR), but the mechanism by which EB causes these actions has not been identified. Further, the properties of EA have not been evaluated. Therefore, the goals of this study were to determine if EA and EB possessed similar activity and if these actions were IGF-IR independent. We utilized synthetic peptides for EA, EB, and a scrambled control to examine cellular responses. Both E-peptides increased MAPK signaling, which was blocked by pharmacologic IGF-IR inhibition. Although the E-peptides did not directly induce IGF-IR phosphorylation, the presence of either E-peptide increased IGF-IR activation by IGF-I, and this was achieved through enhanced cell surface bioavailability of the receptor. To determine if E-peptide biological actions required the IGF-IR, we took advantage of the murine C2C12 cell line as a platform to examine the key steps of skeletal muscle proliferation, migration and differentiation. EB increased myoblast proliferation and migration while EA delayed differentiation. The proliferation and migration effects were inhibited by MAPK or IGF-IR signaling blockade. Thus, in contrast to previous studies, we find that E-peptide signaling, mitogenic, and motogenic effects are dependent upon IGF-IR. We propose that the E-peptides have little independent activity, but instead affect growth via modulating IGF-I signaling, thereby increasing the complexity of IGF-I biological activity.

## Introduction

Insulin-like growth factor-I (IGF-I) is a circulating autocrine/paracrine factor that regulates pre- and postnatal growth in many tissues. Proper embryonic development relies on IGF-I signaling, as IGF-I Receptor (IGF-IR) knockout mice die at birth, and IGF-I knockout mice rarely survive [1]. The IGF-I null mice that do survive have diminished organismal growth [2], whereas mice over-expressing IGF-I systemically are 1.3 times as large as controls [3], indicating that IGF-I signaling is also essential for normal postnatal growth.

IGF-I is one of the major growth factors that directs skeletal muscle development, growth, and regeneration. When IGF-IR is specifically inactivated in skeletal muscle, muscles are 10–30% smaller [4], [5]. Increasing IGF-I in muscle by infusion of recombinant IGF-I [6], transgenic over-expression [7], [8], or viral gene delivery [9], causes hypertrophy, can improve diseased muscle phenotype and function [10], [11], and enhances regeneration after injury [12], [13].

IGF-I activates the conventional pathways of muscle cell proliferation and differentiation in growth and repair [14]. Muscle regeneration relies on a stem cell-like niche of quiescent muscle progenitor cells called satellite cells. Once activated, the satellite cells become myoblasts, proliferate, migrate to the region of injury, and differentiate by fusing with myofibers (reviewed in [15]). IGF-I is upregulated in hypertrophic muscles and after damage or overload [16], [17], and stimulates satellite cells [18]. IGF-I regulates muscle growth via binding to and activating IGF-IR. Upon IGF-I binding, IGF-IR is autophosphorlated at several sites on its cytoplasmic tails, which initiates multiple signaling cascades. Activated IGF-IR triggers the MAPK pathway, increasing proliferation and migration in satellite cells and myoblasts. The PI3-Kinase/Akt pathway is also stimulated, which leads to increased differentiation and protein synthesis in mature muscle fibers [19], [20], [21].

The general consensus is that these growth effects are mediated by mature IGF-I, but the *Igf1* gene encodes more than just the mature growth factor. *Igf1* pre-mRNA is alternatively spliced at the 5′ and 3′ ends, generating multiple isoforms. The *Igf1* gene and its splicing are highly conserved in vertebrates [22]. The pre-proproteins consist of the signal peptide, IGF-I, and a carboxy-terminal extension called the E-peptide [23]. In rodents, there are 2 possible E-peptide extensions: EA and EB. In humans, 3 possible E-peptide extensions have been identified: EA, EB (unique) and EC (like rodent EB) [24]. In all cases, the predominant *Igf1* isoform expressed is *Igf1a*, which is the most conserved across all species examined [22], [24], [25], [26], [27]. Subtilisin-related proprotein convertases (SPCs) can cleave proIGF-I within the constitutive secretory pathway, resulting in mature IGF-I and any of the E-peptides [28], [29], [30]. In addition, IGF-I still connected to the E-peptides (pro-IGF-I) has been found outside of cells, implying that not all of the IGF-I produced is secreted in the mature form, and that the E-peptides can be secreted out of the cell still attached to IGF-I [31], [32], [33]. All isoforms encode the identical mature IGF-I protein, but the E-peptides share less than 50% amino acid identity [34]. For clarity, we will use the rodent terminology, as this study focuses on the rodent isoforms.

The functions of the E-peptides are largely unknown, but focus has been on the less prominent isoforms rather than EA. Much attention has been paid to EB particularly in muscle, where this form has been deemed “Mechano Growth Factor” (MGF) due to rapid transcriptional upregulation of *Igf1b* after stretch, overload, and injury [35], [36], [37], [38]. Exposure to MGF/EB peptides has been shown to increase myoblast proliferation and migration, and overexpression of *Igf1b* delays differentiation [39], [40], [41]. Many of these effects were apparent even when IGF-IR was blocked via a neutralizing antibody, indicating that EB-peptide actions were independent of IGF-I signaling. While MGF/EB has been extensively investigated in muscle growth, EA has been all but ignored, even though 90–95% of the mammalian *Igf1* mRNA transcripts are *Igf1a* [25].

Comparisons of the IGF-I isoforms support that they have both unique and common properties. Increased expression of *Igf1a* and *Igf1b* causes different degrees of hypertrophy in adult mice [42], suggesting that EA and EB act differently *in vivo*. In addition, the presence of either E-peptide enhances the entry of IGF-I into cells [43], showing that they may also share common properties in modulating IGF-I. However, since the *Igf1* gene encodes one E-peptide for every mature IGF-I, and that most of the published functions of the E-peptides are similar to IGF-I actions, it is difficult to discriminate IGF-I and E-peptide effects. In this study, we have utilized synthetic E-peptides to manipulate E-peptide levels independently of IGF-I. The goals of this study are to determine if the E-peptides act independently of IGF-I and IGF-I signaling, and to compare EA and EB biological actions in the model of muscle formation, which includes signaling, proliferation, migration, and differentiation.

## Results

### EA and EB synthetic E-peptides enhance MAPK signaling

**Figure 1:**
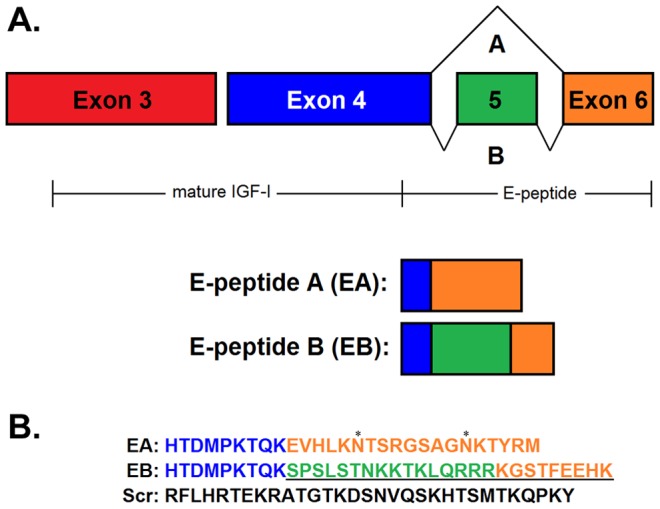
Synthetic E-peptide sequences. **A**. Rodent *Igf1* 3′ splicing leads to two mRNA isoforms. While mature IGF-I is encoded by exons 3 and 4, the E-peptides are encoded by exons 4, 5, and/or 6. EA isoforms exclude exon 5, while EB isoforms retain exon 5, leading to an altered reading frame and earlier stop codon in exon 6. Exons not drawn to scale **B**. Synthetic E-peptide amino acid sequences. EA and EB are less than 50% identical. Scr = Scrambled peptide. * = potential glycosylation sites in EA. The portion of EB that corresponds to MGF is underlined.

IGF-I is known to activate the MAPK and PI3-Kinase/Akt pathways in many cell types including skeletal muscle myoblasts [13], [14], [44]. If the E-peptides work similarly to, or in concert with IGF-I, they may also affect these signaling pathways. Indeed, previous studies have examined the effects of the E-peptides in the MAPK signaling cascade and have observed that synthetic MGF peptide increased ERK phosphorylation in rat cardiomyoblasts [45], and in mouse skeletal muscle myoblasts [46]. However, EA has never been evaluated for signaling effects. To compare EA and EB in C2C12 mouse myoblast culture, synthetic E-peptides were generated (Fig. 1). The synthetic peptides begin immediately following the SPC site in exon 4. They include the C-terminal portion of exon 4, plus exon 6 (EA) or exons 5 and 6 (EB) (Fig. 1 A). In contrast to the MGF peptide used in previous studies, which included only the unique portion encoded by exons 5 and 6, the more biologically relevant EB peptide we generated includes additional residues that would be retained following cleavage of pro-IGF-I. A scrambled (Scr) peptide was also generated as a negative control.

**Figure 2:**
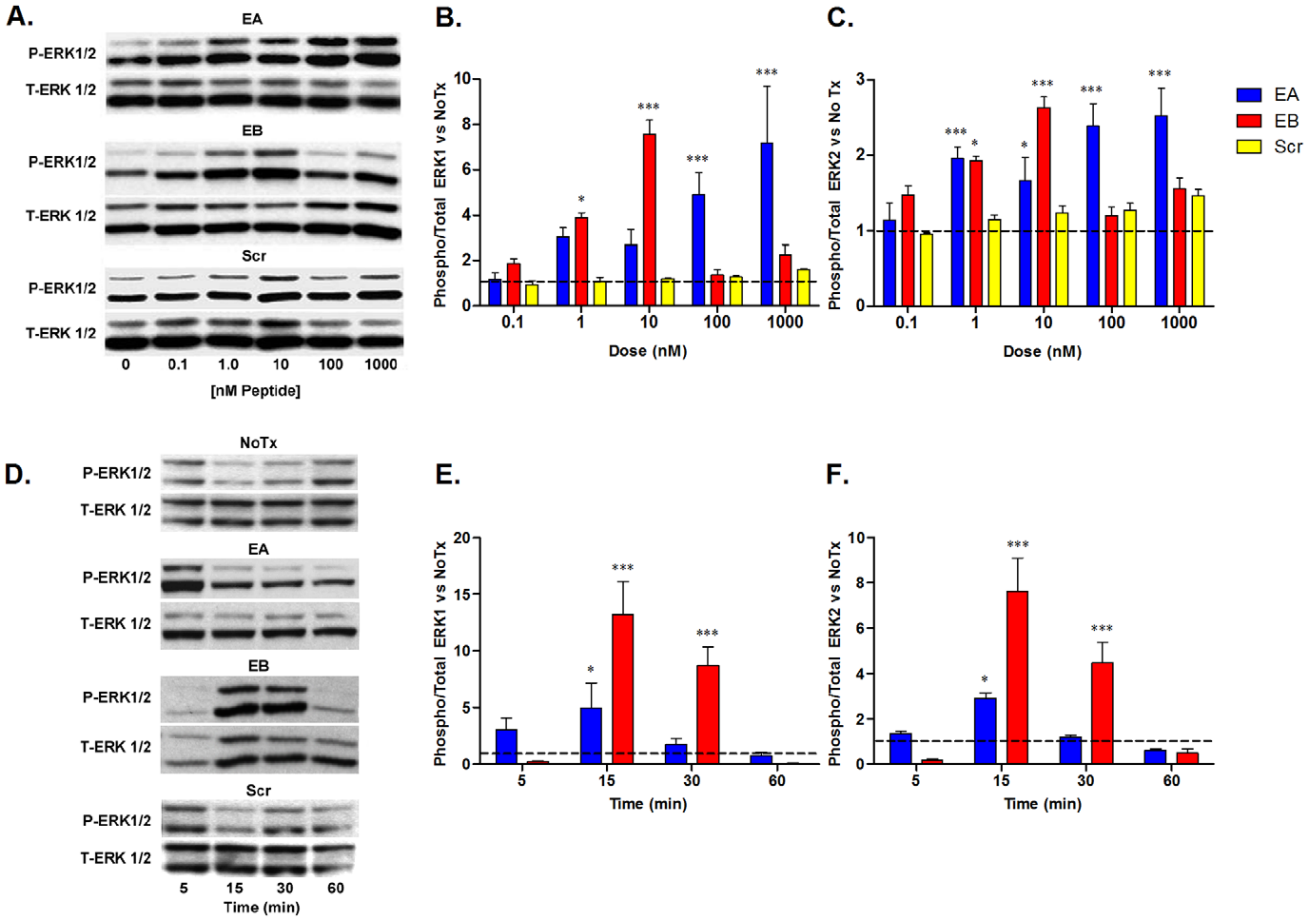
EA and EB increase MAPK signaling in C2C12 cells. **A**. Cells were starved in media without serum, and treated with synthetic E-peptides at concentrations indicated for 20 minutes. Protein lysates were separated via SDS-PAGE and immunoblotted for Phosphorylated ERK 1 and 2 (P-ERK1/2), stripped, and blotted for Total ERK 1 and 2 (T-ERK1/2). **B–C**. Quantification of A. **D**. Cells were treated as above at optimal doses (EA and Scr 1 µM, EB 10 nM) for times indicated. **E–F** Quantification of C. NoTx at 30 minutes was included in each experiment for normalization between blots. For B–C and E–F, bars represent means ± s.e.m. of N = 3 replicates. *, p<0.05; ***, p<0.001, for comparisons to NoTx via 2-way ANOVA followed by a Bonferroni post-test.

To compare E-peptide effects on signaling, C2C12 cells were exposed to synthetic E-peptides. Multiple signaling pathways were initially examined (SMAD, p38, Jnk, and Akt), but only the MAPK pathway was affected by E-peptide exposure. To determine the response to E-peptides, immunoblotting for phosphorylated and total ERK1/2 was performed following treatment with increasing concentrations of EA, EB, or Scr (Fig. 2 A–C). Increased ERK1/2 phosphorylation was evident with E-peptide concentrations as low as 1 nM. EA exhibited dose-dependent signaling. EB was more potent, with significantly higher P-ERK1 at 1 nM compared to untreated cells (NoTx, DMEM without serum, IGF-I, or E-peptides). The maximum response to EB occurred at 10 nM, with diminished phosphorylation at higher concentrations (100 nM–1 µM). ERK1 had higher activation than ERK2 at optimum dose for both EA and EB, with 8 times more phosphorylation than NoTx for ERK1, and 3 times more for ERK2. Scr did not increase P-ERK 1/2 significantly at any concentration, and thus the P-ERK1/2 responses were specific to each sequence, and not to a random peptide.

Next, the synthetic E-peptides were used at concentrations generating the optimum response (EA and Scr 1 µM, EB 10 nM) to examine the time course of ERK1/2 phosphorylation (Fig. 2 D–F). Cells treated with DMEM only (NoTx) were also collected at times indicated to obtain the time-course baseline of ERK1/2 phosphorylation. EA treatment showed an increase in P-ERK1/2 early (5 min), but because NoTx cells had a high basal ERK1/2 phosphorylation at 5 minutes, the difference was not significant after quantification. Both E-peptides generated a transient increase in P-ERK1/2. EA-induced P-ERK1/2 reached maximum levels by 15 minutes after E-peptide addition, but fell to untreated levels by 30 minutes. In contrast, the P-ERK1/2 response to EB was not detectable until 15 minutes after exposure, but remained elevated for at least 30 minutes. Exposure to Scr did not cause any increase in P-ERK1/2 at any time point. Thus, both E-peptides can transiently increase ERK1/2 phosphorylation at their optimum concentrations; however, EB is active at lower concentrations for a more sustained period.

### E-peptide signaling depends on the IGF-I receptor

**Figure 3:**
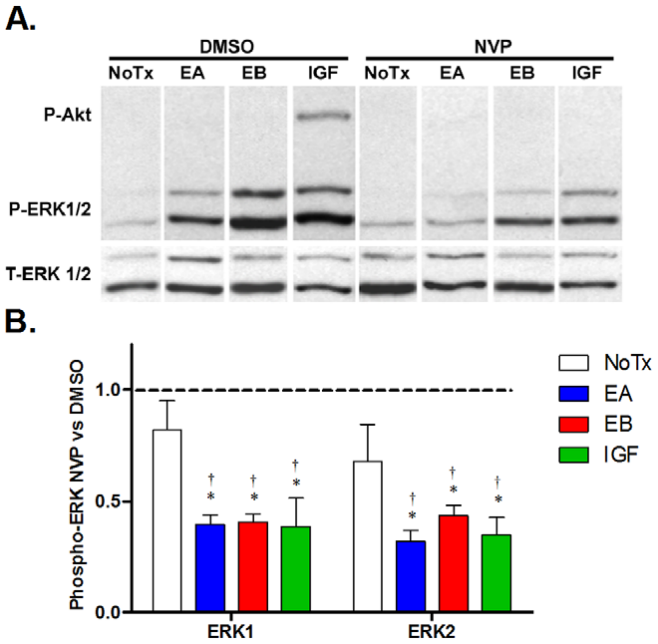
E-peptide ERK signaling requires IGF-IR. **A**. C2C12 cells were treated as in Figure 2, except the IGF-IR inhibitor NVPAEW541 (NVP, 100 nM) or DMSO was added to cells 90 minutes prior to and during stimulation for 20 minutes. Cells were treated with EA (1 µM), EB (10 nM), or recombinant IGF-I (IGF, 10 nM) as a positive control. EA and EB experiments were conducted separately, and image represents one sample from each experiment. **B**. Quantification of A. Data are presented as the effect of NVP on ERK phosphorylation: (P/T-ERK of NVP treated)/(P/T-ERK of DMSO treated cells). NoTx bands were used to compare between experiments. Bars represent means ± s.e.m. of N = 3 replicates. *, p<0.05 for comparisons of treatments to NoTx via 1-way ANOVA followed by a Tukey post-hoc test; †, p<0.05 for comparisons of NVP samples to their DMSO counterparts via student t-tests.

Although IGF-I and the E-peptides are produced by the same gene, there is no known functional relationship between them; however, because they share the ERK1/2 phosphorylation response, it is possible that the E-peptides enhance MAPK signaling cooperatively with IGF-I via the IGF-IR. To determine if the E-peptide signaling effects were dependent upon IGF-I signaling, pharmacologic inhibition of the IGF-IR was utilized in conjunction with E-peptide exposure. NVPAEW541 (NVP), a small molecule inhibitor of IGF-IR tyrosine kinase activity [47], was utilized in combination with IGF-I or the E-peptides. Treatment with IGF-I caused an increase in both P-Akt and P-ERK1/2, and these responses were blocked in the presence of NVP (Fig. 3 A,B) confirming that NVP effectively inhibited IGF-IR signaling. Using the E-peptide concentrations shown in Fig. 2, both EA and EB caused increased P-ERK1/2, but no change in P-Akt (Fig. 3 A, lanes 1–3). Interestingly, in the presence of NVP, the P-ERK1/2 response was ablated in EA and EB treated cells to a similar extent as in IGF-I treated cells (Fig. 3 B). There was no change in ERK1/2 phosphorylation in NoTx cells with or without NVP, which verified that NVP was neither harmful to the cells nor affected IGF-I-independent MAPK signaling. However, NVP significantly reduced the ERK1/2 phosphorylation in EA, EB, and IGF-I treated cells, establishing that a functional IGF-IR is required for E-peptide induced ERK1/2 activation.

### E-peptides enhance IGF-IR activation by IGF-I through increasing receptor cell surface bioavailability

**Figure 4:**
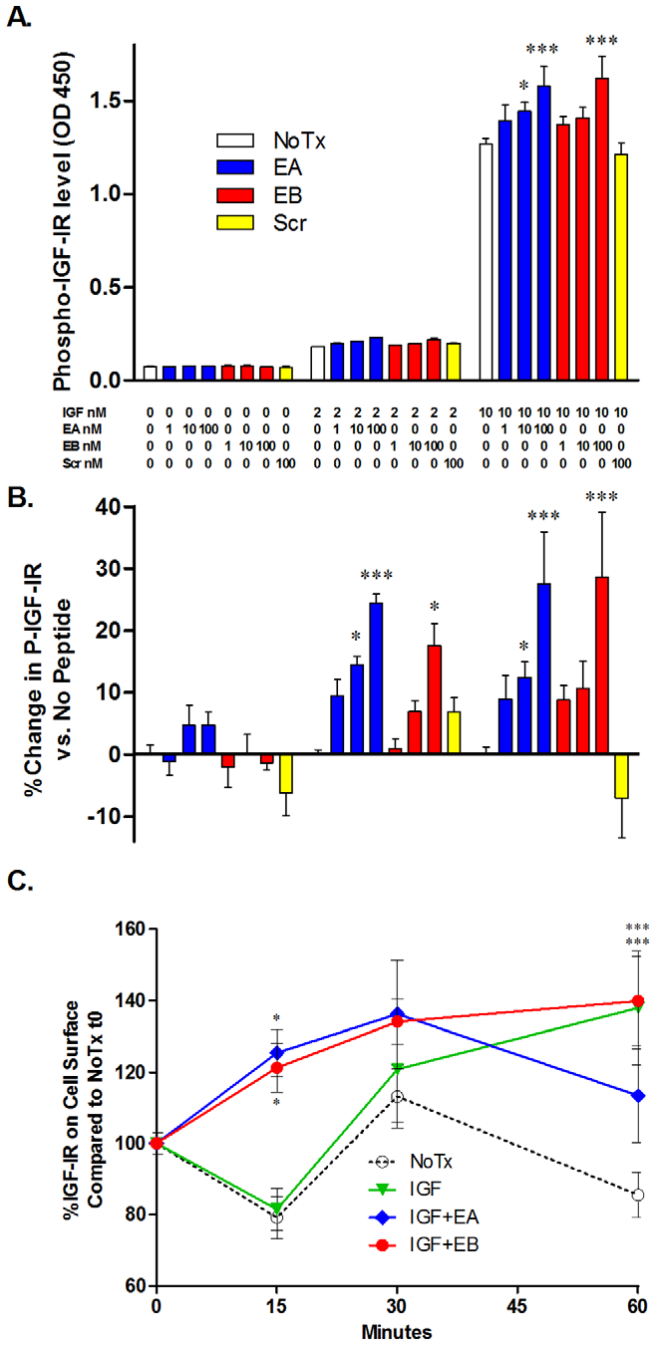
E-peptides augment IGF-IR activation and cell surface localization. **A**. P6 cells overexpressing IGF-IR were treated with synthetic E-peptides with and without recombinant IGF-I for 15 minutes, and cell lysates were utilized for KIRA assays. Level of absorbance indicates the extent of IGF-IR phosphorylation. Bars represent means ± s.e.m. of N = 6 wells. **B**. OD 450 from A were compared to No Peptide for each IGF-I concentration, and the % change is graphed. **C**. P6 cells were treated as in A for a localization assay for times indicated, and biotin labeled before lysis. The optimal concentrations of E-peptides and IGF-I from the A were used (E-peptides 100 nM, IGF-I 10 nM). Surface IGF-IR was normalized to Total IGF-IR and compared to NoTx at t0 to get % IGF-IR on cell surface. Bars represent means ± s.e.m. of N = 6 wells. Samples were compared to no peptide (A and B,*), NoTx (C,*) or IGF-I (C,†) via 2-way ANOVA followed by a Bonferroni post-test. * or †, p<0.05; ***, p<0.001.

The IGF-IR dependence of E-peptide signaling could be due to direct interaction of the E-peptides with the receptor, or through an indirect mechanism with the IGF-I ligand. To test IGF-I dependent and independent IGF-IR activation by the E-peptides, a kinase receptor activation (KIRA) assay was performed. This assay utilizes IGF-IR over-expressing mouse fibroblasts (P6 cells) [48], [49] to provide sufficient receptor density for detection. IGF-I alone stimulated IGF-IR phosphorylation dramatically and significantly at 2 nM and 10 nM. In the absence of IGF-I, cells treated with EA, EB, or Scr showed no evidence of receptor activation (Fig. 4 A,B). However, combined exposure of IGF-I and either EA or EB significantly increased IGF-IR activation compared to IGF-I alone. EA at 10 and 100 nM significantly increased IGF-IR activation compared to no peptide in the presence of 2 or 10 nM IGF-I. EB was less potent, significantly increasing IGF-IR phosphorylation at 100 nM with IGF-I at 2 and 10 nM. Scr at 100 nM was used as a negative control, and it did not increase IGF-IR phosphorylation at any concentration of IGF-I. These results indicate that although the E-peptides do not activate IGF-IR directly, they augment IGF-IR activation in an IGF-I-dependent manner.

The lack of direct E-peptide activation of IGF-IR in combination with enhancement of ligand-mediated activation suggests that the E-peptides could modulate the availability of receptors for IGF-I. To examine this, cell-surface proteins on P6 cells were biotin labeled after treatment with the E-peptides and/or IGF-I for 15, 30, and 60 minutes, and cell-surface IGF-IR was compared to total IGF-IR. Overall, IGF-I and NoTx showed the same pattern of internalization from 0–30 minutes, with an accumulation of recycled cell surface receptors [50] in IGF-I treated cells by 60 minutes. However after 15 minutes, the time point used in the KIRA assay, stimulation with IGF-I and either E-peptide caused a significant increase in the proportion of IGF-IR on the cell surface compared to NoTx and IGF-I alone (Fig. 4 C). Thus, one mechanism for augmented IGF-IR activation after E-peptide stimulation is that the E-peptides increase the bioavailability of IGF-IR for its ligand, IGF-I, by increasing cell surface IGF-IR.

### E-peptides enhance IGF-IR downstream signaling

**Figure 5:**
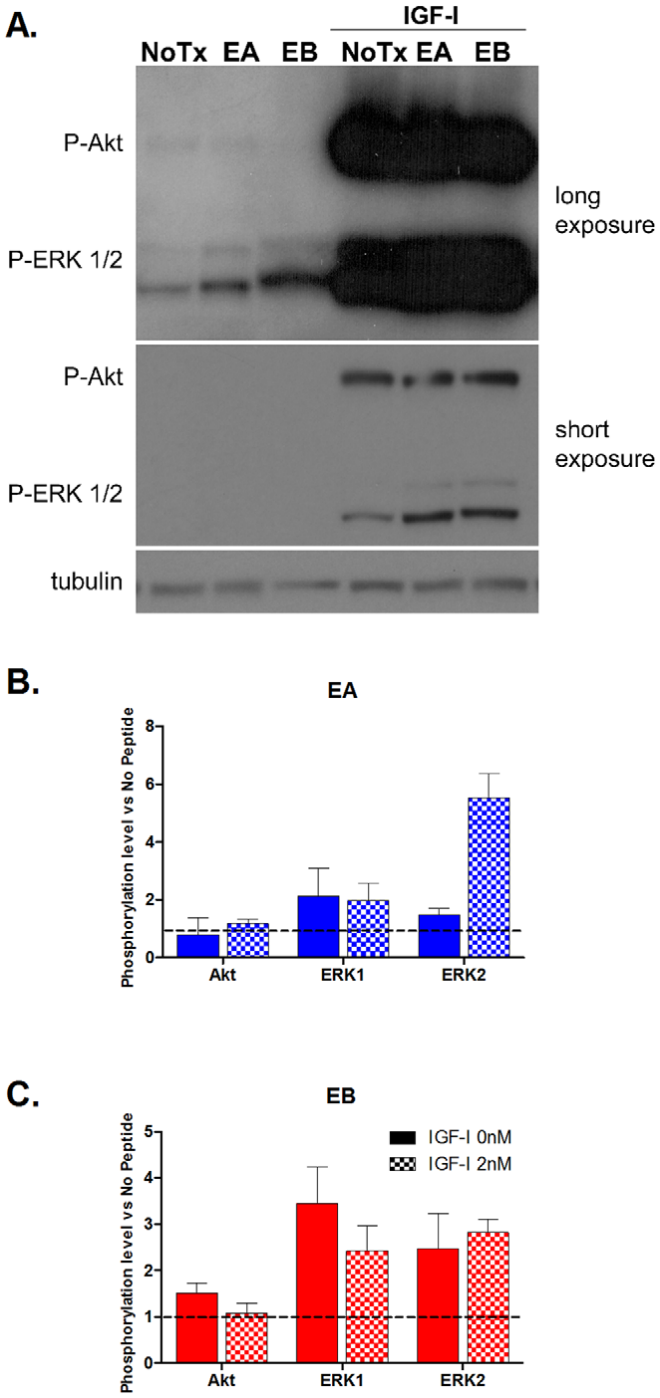
E-peptides affect IGF-IR downstream signaling. **A–C**. C2C12 cells were treated as in Figure 2, with 0 nM or 2 nM IGF-I and optimal doses of the E-peptides (EA 1 µM, EB 10 nM) for 20 minutes. **B–C**. Quantification of Akt and ERK1/2 phosphorylation after EA (B) or EB (C) treatment. EA and EB alone are compared to NoTx, while IGF-I with E-peptides are compared to 2 nM IGF-I alone. Data are presented as the effect on phosphorylation after E-peptide treatment compared to NoTx or NoPeptide plus IGF-I. Bars represent means ± s.e.m. of N = 3–4 replicates. †, p<0.05 for comparisons of 0 nM IGF samples to their 2 nM IGF-I counterparts via student t-tests.

Since we observed that the E-peptides augment IGF-IR signaling in P6 cells (Fig. 4), and increase MAPK signaling but not Akt phosphorylation in myoblasts (Fig. 2), we predicted that the E-peptides might differentially activate a subset of IGF-IR mediated pathways. To test if the E-peptides could alter MAPK or Akt/PI3Kinase pathways after IGF-I stimulation in myoblasts, C2C12 cells were stimulated with the E-peptides alone, IGF-I alone, or IGF-I plus EA or EB. Consistent with our previous signaling experiments, without IGF, the E peptides increased P-ERK1/2 approximately 2–4 fold compared to NoTx (0 nM IGF-I), and there was no change in P-Akt. In the presence of 2 nM IGF-I, there was also a 2–4 fold increase in P-ERK1/2 with the addition of the E peptides compared to IGF-I alone (Fig. 5 A–C). Clearly, the E-peptide enhancement of IGF-IR phosphorylation led to altered IGF-IR downstream signaling, which favored MAPK without altering Akt/PI3Kinase pathways. In addition, the enhancement in P-ERK2 after IGF-I and EA treatment compared to IGF-I alone was significantly higher than the enhancement after EA vs NoTx, indicating that EA may augment IGF-I potency.

### E-peptide effects on myoblast proliferation and migration are IGF-IR dependent

**Figure 6:**
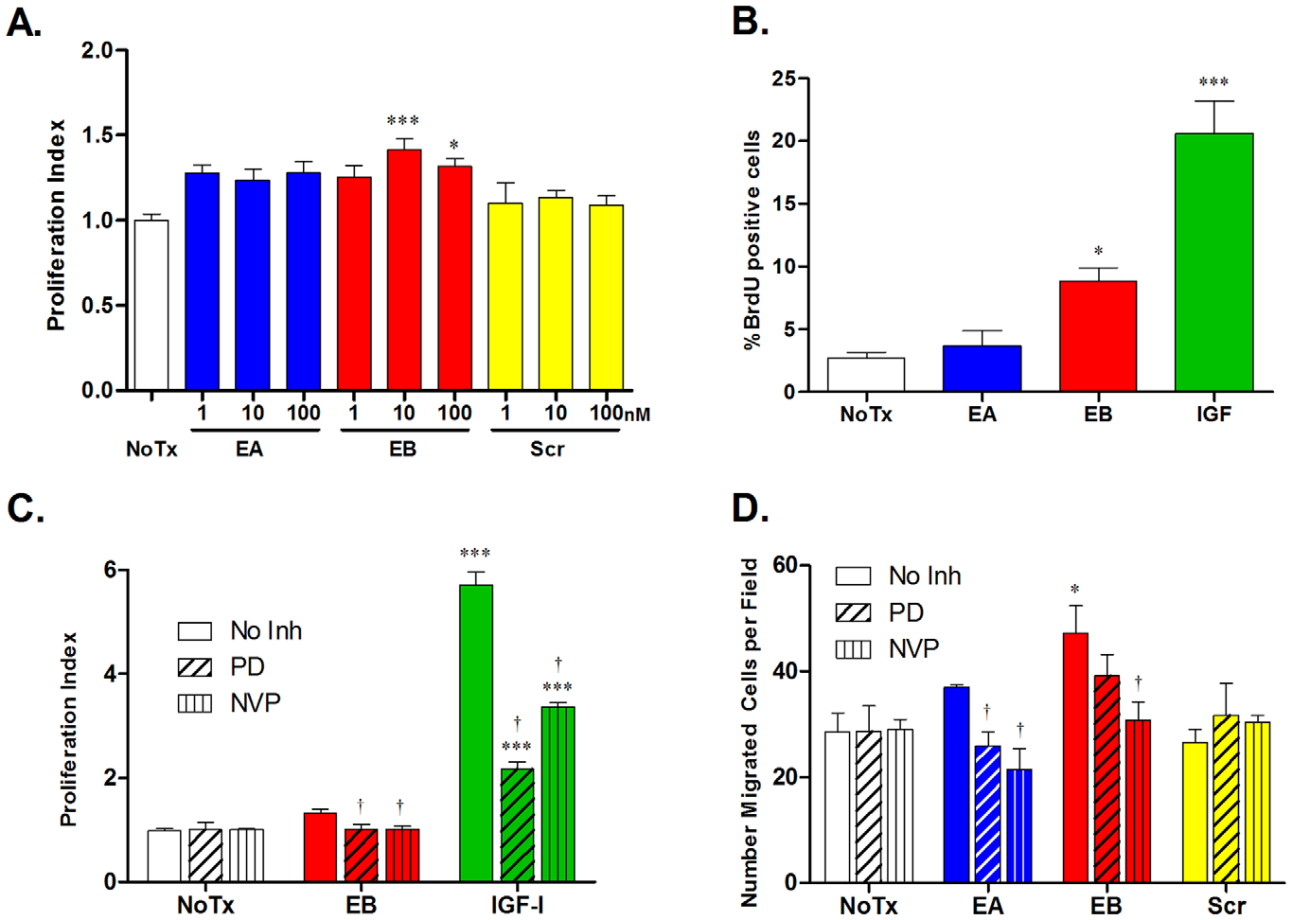
EB increases in myoblast proliferation and migration are MAPK and IGF-IR signaling dependent. **A**. C2C12 cells were plated in 96 well plates, starved for 6 hours, and treated with synthetic E-peptides. A BrdU plate assay was used to quantify proliferating cells, where increased absorbance is correlated with increased proliferation. Bars represent means ± s.e.m. of N = 10 wells. **B**. A similar BrdU assay was used to visualize the proliferating cells on slides. Cells were treated as above (EA and Scr 100 nM, EB and IGF 10 nM), fixed, and stained with BrdU and DAPI. Total cells and proliferating (BrdU positive) cells were counted from 3 10× fields for each slide, and bars represent means ± s.e.m. of N = 5 slides. For A and B, *, p<0.05; ***, p<0.001 for comparisons to NoTx via 1-way ANOVA followed by a Tukey post-hoc test. **C**. C2C12 cells were tested as in A, except an inhibitor of MEK, PD 098059 (PD, 50 µM) or IGF-IR (NVP, 100 nM) was included in the cell media. Bars represent means ± s.e.m. of N = 18 wells for No Inhibitor (No Inh) and N = 8 for with inhibitors. **D**. C2C12 cells were plated in the upper chamber of 24-well plate trans-well migration inserts in 0% serum media. Cells were allowed to migrate for 5 hours and stained with DAPI, imaged and counted. Synthetic E-peptides (100 nM) were added to upper and lower chambers with or without inhibitors (PD 50 µM, NVP 100 nM). Images were taken as in B, and bars represent means ± s.e.m. of N = 4 slides. For C and D, *, p<0.05; ***, p<0.001 for comparisons to NoTx via 2-way ANOVA followed by a Bonferroni post-test. †, p<0.05 for comparisons to No Inh via 2-way ANOVA followed by a Bonferroni post-test.

To examine the importance of IGF-IR for the biological actions of the E-peptides, we utilized the model of skeletal muscle growth in cell culture, which has been studied extensively with IGF-I and MGF [39], [51], [52]. First, we focused on myoblast proliferation, which increases in the presence of IGF-I [14] and MGF/EB [39], [40], [53], [54], [55], [56], but neither EA nor full-length EB have been evaluated in myoblasts. Therefore, we determined the effects of EA, EB, and Scr on myoblast proliferation. We examined C2C12 proliferation in an ELISA plate assay for BrdU after treatment with the synthetic E-peptides at different concentrations. While both EA and EB treated cells showed a modest trend towards increased proliferation at all concentrations, only 10 and 100 nM EB increased proliferation significantly, by approximately 35–40% (Fig. 6 A). To directly observe the proliferating cells, C2C12 cells were grown on cover slips and examined for BrdU positivity using fluorescent microscopy. Recombinant IGF-I treatment was used as a positive control. EB and IGF-I increased C2C12 proliferation, but EA did not (Fig. 6 B).

To clarify if the proliferative effects of EB were mediated by the MAPK pathway or dependent upon IGF-IR, cells were exposed to the optimum concentration of EB (10 nM), with or without pharmacologic inhibition of MEK, a MAP kinase upstream of ERK, by PD 098059 (PD) [57], or by NVP to inhibit IGF-IR activity (Fig. 6 C). IGF-I (10 nM) was used as a positive control. PD and NVP significantly decreased IGF-I induced proliferation, confirming that IGF-I mediates these effects predominantly through the MAPK pathway and IGF-IR. EB increased proliferation significantly without inhibitors, but these effects were blocked in the presence of PD or NVP. Therefore, EB requires MAPK signaling and a functional IGF-IR to increase myoblast proliferation.

In skeletal muscle growth and repair, after myoblasts proliferate, they must migrate to the areas in need of extra nuclei. Myoblast migration has been linked to MAPK signaling activation [58]. Accordingly, the increase in ERK1/2 phosphorylation after E-peptide stimulation could lead to increased myoblast migration. To determine if the E-peptides modulate migration, and if the effects are MAPK or IGF-IR dependent, a trans-well assay using serum-starved C2C12 cells was used (Fig. 6 D). Only EB treatment caused enhanced myoblast migration, with a 70% increase in the number of migrating cells, whereas EA treated cells showed ∼30% enhancement that was not significantly different from untreated cells. Cell migration in the absence of E-peptides was not affected by either PD or NVP, most likely due to the brief duration of the experiment in the absence of serum, and thus an absence of endogenously secreted growth factors. Blockade of MAPK signaling by PD caused a decrease in EB migration that did not reach significance, suggesting that EB mediates migration via pathways in addition to MAPK. Blockade of IGF-IR activation by NVP significantly decreased EB induced migration, indicating that EB requires IGF-IR to increase myoblast migration. Interestingly, with EA, there was not a significant increase in migration without inhibitors, yet both inhibitors decreased migration significantly when EA was present. In sum, migration driven by the E-peptides, especially EB, is dependent upon the IGF-IR.

### Myoblast differentiation is inhibited by E-peptides

**Figure 7:**
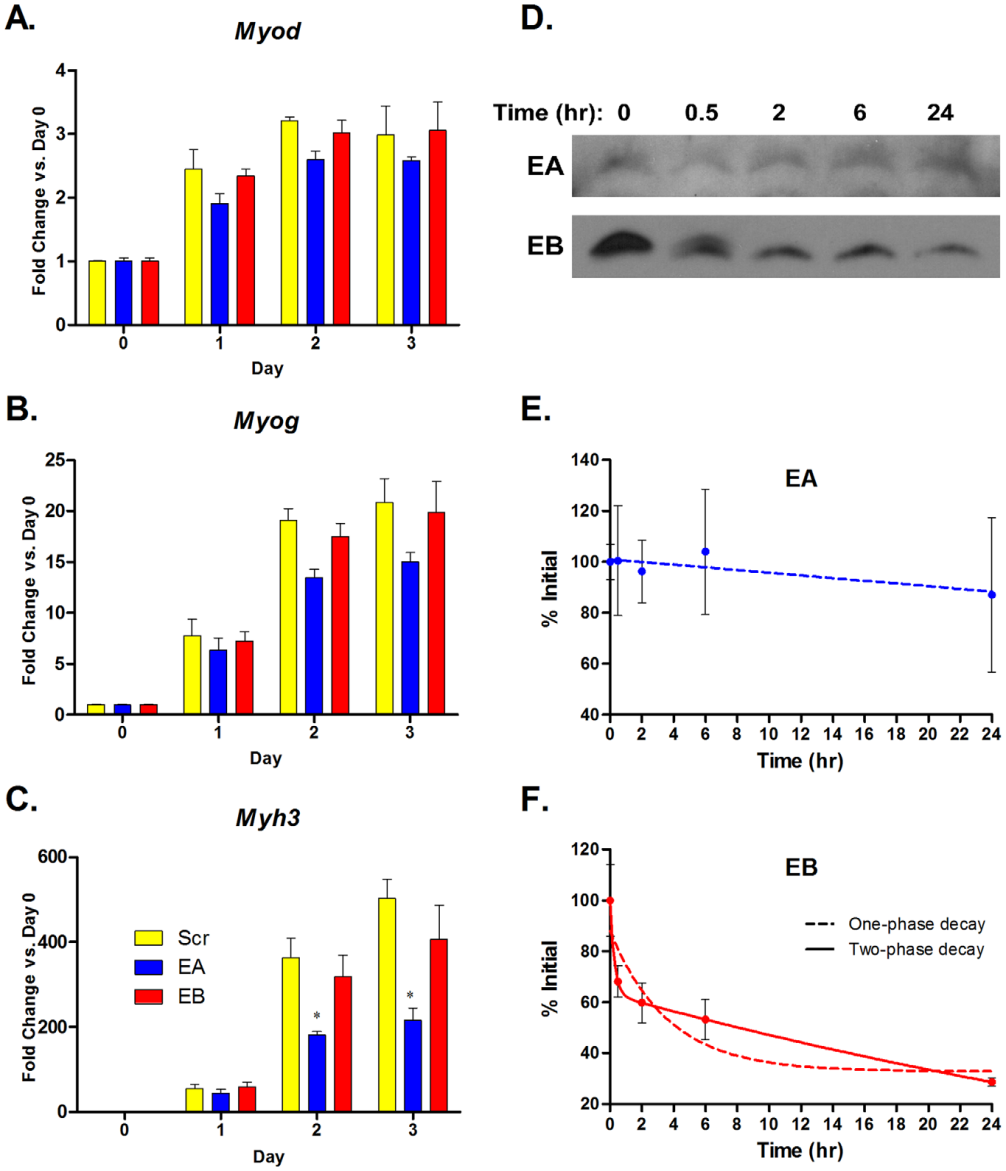
E-peptides inhibit myoblast differentiation. **A–C**. C2C12 cells were grown to confluency and switched to differentiation media (Day 0). Media was changed every day and synthetic peptides (100 nM) were added to the fresh media. Quantitative RT-PCR was used to measure expression of differentiation markers: MyoD (*Myod*, A), Myogenin (*Myog*, B), Embryonic Myosin (*Myh3*, C). Expression of the markers at Days 1, 2, and 3 were compared to Day 0 to obtain fold change. Bars represent fold change means ± s.e.m. of N = 3 replicates. *, p<0.05 for fold change expression comparisons via 2-way ANOVA followed by a Bonferroni post-test. **D**. Synthetic EA and EB peptides were incubated with growth media and aliquots were taken at times indicated for immunoblotting analysis. **E–F**. Quantification of (D) and analysis of peptide half-life. Bars represent percent of original intensity ± s.e.m. of N = 3 replicates.

Skeletal muscle growth and repair rely on satellite cells and myoblasts to fuse with existing muscle fibers or with each other to differentiate into new muscle fibers. This process can be replicated in culture to examine key markers of differentiation and myotube formation. Previous studies showed that MGF/IGF-IB delays myoblast differentiation [39]. To compare the effects of EA and EB on differentiation, we treated differentiating C2C12 cells with EA, EB, or Scr synthetic peptides for three days and used qRT-PCR to evaluate changes in expression of differentiation markers MyoD (*Myod*), Myogenin (*Myog*), and Embryonic myosin (*Myh3*) (Fig. 7 A–C). All three markers increased as days of differentiation increased. There was no significant difference between treated cells at any day for *Myod* or *Myog*, however, there was significantly less *Myh3* expression in EA treated cells versus Scr treated cells at both Day 2 and 3. EB treated cells showed a trend towards lower *Myh3* expression, but it did not reach significance. Thus EA impaired the later stages of differentiation and maturation.

In this study, EB did not inhibit differentiation, although MGF has been shown to delay differentiation [39]. This may be because EB, which has a potential protease cleavage site, is not stable in cell media for long enough to consistently affect differentiation. To determine the half-life of the E-peptides, synthetic EA and EB were incubated in growth media containing 10% FBS, and aliquots were taken for immunoblotting at various time points (Fig. 7 D–F). While EA showed no signs of instability, EB clearly and quickly degraded. In fact, it is apparent that EB after 2 hours was a smaller size than the initial peptide, and the peptide at 30 minutes appears as a doublet. We believe that the full-length peptide is cleaved at the potential cleavage site located at the exon 5/6 boundary. A 2-phase exponential decay showed that the half-life of the full-sized peptide is 20 minutes, and the half-life of the remaining peptide is approximately 40 hours. So, EB may delay differentiation, but synthetic EB cannot be tested in our assay due to instability.

## Discussion

Previous reports contend that the C-terminal E-peptide of the rodent *Igf1b* gene splice form harbors an additional growth factor, MGF/EB, that acts independently of IGF-I. We challenged this hypothesis by comparing both C-terminal E-peptides, EA and EB, and by using a pharmacologic approach to decrease IGF-IR activity. Similar to past studies, we observed increased MAPK signaling that was concentration dependent and specific to the E-peptides. However, in contrast to those findings, we demonstrated that these signals were dependent upon the availability of IGF-IR. We extended this observation to determine if this was through direct activation of the receptors, which was not the case. Instead, the presence of the E-peptides significantly enhanced IGF-I mediated receptor phosphorylation, in part through increasing the proportion of receptors on the cell surface. By increasing the available pool of receptors for ligand binding, there was greater receptor activation. Next, we evaluated the IGF-I dependent and independent effects of rodent IGF-I E-peptides on the cellular processes necessary for muscle formation. We found that both E-peptides are mitogens in skeletal muscle cell culture, and that EB is more potent in driving proliferation and migration compared to EA. Not only do these actions require the MAPK pathway, E-peptide activity is also dependent upon IGF-IR signaling. Hence, in contrast to previous studies demonstrating IGF-I independent actions of MGF/EB, we find that the E-peptides coordinate with IGF-I at several key points during muscle growth. In fact, all of the E-peptide effects we have observed depend upon IGF-I and IGF-IR. We have now excluded independent activity through pharmacologic ablation of IGF-I receptor activity, and suggest that the E-peptides work in concert with IGF-I. Thus, we assert that the E-peptides are not independent growth factors, but instead are modulators of IGF-I actions.

To examine the effects of E-peptides, we synthesized peptides that reflected products of the *Igf1* gene. Synthetic rodent MGF, which has been tested in previous studies, contains the last 25 amino acids in IGF-IB beginning at the exon 4/5 boundary (Fig. 1 A and B), so it shares no sequence homology with EA. While these residues may contain the active domains of EB, there is no evidence that MGF at this size exists *in vivo*, since there are no known cleavage recognition sequences between Exons 4 and 5 [34], [59]. For these reasons, we included the residues encoded by Exon 4 in both EA and EB to mimic the predicted processing of the IGF-I proprotein [28]. Even so, the effects of synthetic EB are similar to those with MGF [39], [40], and so it is likely that the N-terminal portion of EB is dispensable in terms of activity. Both E-peptide sequences contain a high percentage of polar and basic amino acid residues. To eliminate the possibility that the effects we observed were simply due to the presence of a charged peptide, we generated a random sequence, Scramble (Scr), based on the average charge and residue content of both E-peptides. Because Scr did not affect any of the processes we studied, even up to 1 µM, we are confident that the changes we observed after exposure to EA or EB were due to the specific sequences within these peptides.

While the synthetic E-peptides afford evaluation of their activity at exact concentrations and durations, there are some biological drawbacks. For example, the rodent EA peptide contains two potential glycosylation sites (* in Fig. 1 B). Synthetic EA does not have glycosylated asparagine residues. If the glycosylation on EA is important for its mitogenic actions, then we will not see all of EA's biological activity. An additional issue is that the instability of EB, which has a potential protease cleavage site at the exon 5/6 boundary, may abbreviate its activity [34]. In fact, we found that the half-life of full-length synthetic EB is only 20 minutes, which may explain why we did not observe differentiation effects by EB over the course of a 3-day experiment (Fig. 7). However, the shorter EB form likely has some mitogenic activity, as EB affected proliferation over a 24-hour treatment period, and enhanced ERK1/2 phosphorylation up to 30 minutes after treatment. Alternate strategies to prevent degradation by replacing residues in the cleavage site can prolong synthetic EB half-life, and enable longterm studies of its actions. For example, human myoblast senescence was delayed by modified MGF/EB [60]. Nevertheless, the actions *in vivo* are likely curtailed by protease disruption of the peptide.

To our knowledge, we are the first to show that E-peptides augment IGF-IR activation by IGF-I, alter IGF-IR localization, and that E-peptide biological actions are dependent on IGF-I receptor activity. Both E-peptides increased IGF-IR phosphorylation and downstream MAPK signaling, but the E-peptides cannot increase ERK phosphorylation, proliferation, or migration when IGF-IR is inhibited. The E-peptides require a functional IGF-IR, but do not directly activate this receptor alone: IGF-I is required for the E-peptides to increase IGF-IR phosphorylation. Having excluded a direct E-peptide/IGF-IR activation mechanism, we addressed how this activation enhancement might occur through other means. One clue arises from the pattern of signaling following E-peptide exposure. E-peptides alone increase MAPK signaling, but not the Akt/PI3Kinase signaling arm. Because receptor internalization leads to increased P-ERK1/2 [61], the E-peptides may affect receptor internalization, thereby increasing the MAPK arm of the IGF-IR pathway. Consistent with this possibility, we previously observed that the presence of E-peptides enhanced IGF-I uptake (an indicator of receptor internalization) in myoblasts [43]. We thus examined the localization of the IGF-IR on E-peptide and IGF-I treated cells. Instead of finding decreased cell surface IGF-IR, an indicator of receptor internalization, we found that the E-peptides upregulated the proportion of IGF-IR on the cell surface. Consistent with the ∼30% increase in IGF-IR phosphorylation, we observed a ∼40% increase in cell surface IGF-IR after E-peptide treatment, compared to IGF-I alone. Increased surface receptor raises the amount of receptor available for IGF-I binding and activation. Thus, one mechanism by which the E-peptides augment IGF-IR phosphorylation is by increasing the bioavailability of the IGF-IR.

Additionally, when EA or EB and IGF-I were used to stimulate myoblasts, the E-peptides amplified phosphorylation of ERK1/2, but not Akt. In addition, there was a significant enhancement of P-ERK2 between EA+IGF-I vs. EA alone. This effect is consistent with the KIRA results shown in Figure 4, where IGF-IR phosphorylation by IGF-I was enhanced by 30% with the addition of E-peptides, and supports that the effects of the E-peptides occur through the IGF-IR. However, we cannot exclude the possibility that the E-peptides also work indirectly to enhance P-ERK1/2 through and IGF-IR independent mechanism. It should be noted that the MEK inhibitor blocked the E-peptide effects on migration and proliferation, confirming that if the E-peptides act downstream of the IGF-IR, it is above MEK. Regardless of where the E-peptides affect IGF-IR signaling, enhancement of the MAPK arm but not the Akt/PI3K arm suggests that the E-peptides may help to tune the IGF-IR signaling cascade towards MAPK. The concept of receptor tuning has been shown in previous work. For instance, the level of receptor ubiquitination shifts not only its internalization but also the level of MAPK activation [62], [63]. Further, specific regions of IGF-IR have been shown to be important for MAPK but not Akt/PI3K pathways [64], and an IGF-IR growth inhibitor has been found to activate ERK signaling through the IGF-IR, but not Akt [63]. Increased IGF-IR bioavailability on the cell surface leading to increased IGF-IR phosphorylation cannot explain the tuning of the receptor. However, if the E-peptides increase cell surface IGF-IR via enhancing the rate of IGF-IR recycling to the cell surface after internalization, it is possible that the E-peptides accelerate receptor internalization, thus tuning IGF-IR signaling. More direct methods of monitoring receptor trafficking could be used in future studies to track the kinetics of receptor movement.

Several studies have utilized IGF-IR neutralizing antibodies to block IGF-I signaling, and found that MGF can increase proliferation independently of IGF-IR [39], [41], [65]. Neutralizing antibodies bind to the IGF-I recognition site on an extracellular domain of IGF-IR, and block IGF-I from binding to and activating its receptor. They can also, however, lead to receptor internalization and degradation [61], [66]. This can activate or change the localization of IGF-IR, which confounds the interpretation of the results, especially since we found that IGF-IR localization changes in the presence of E-peptides. To avoid these problems, we used an IGF-IR kinase inhibitor, NVPAEW541 (NVP) [47]. NVP blocks the tyrosine kinase autophosphorylation that occurs on the intracellular portion of IGF-IR after ligand binding. By using this inhibitor, IGF-IR localization, levels, and basal signaling do not change, and IGF-I binding is unaffected. These methodological differences may underlie our ability to detect the E-peptide dependence on IGF-IR for signaling, proliferation, and migration.

A second objective of this study was to compare EA and EB, since EA has never been studied. We took advantage of previous demonstrations that MGF/EB affects myoblast signaling, proliferation, migration, and differentiation. It was completely unknown if EA shared or opposed any of MGF/EB's effects. Previous studies have shown that MGF/EB increased ERK1/2 phosphorylation [45], [46], [55]. EB treatment increased ERK1/2 phosphorylation at low concentrations, but ceased to have activity at concentrations 100 nM and higher. This pattern is not unusual, however, as other factors have been known to stimulate MAPK signaling in a bimodal fashion [67]. Our signaling results show that while EB is more potent at activating ERK1/2 both in dose and duration, EA also increases P-ERK1/2. In addition, both EA and EB increased IGF-IR phosphorylation, localization, and downstream signaling when IGF-I was present. Thus, this study demonstrates clear overlap in the actions of the E-peptides.

One limitation to studying the E-peptides is that they have only been detected *in vivo* as part of pro-IGF-I [31], [32], [33], and so attributing any biological activity to the E-peptides independent of IGF-I has been met with skepticism. Although it is unknown at what concentration the free E-peptides are found, one can estimate their levels based on IGF-I concentrations. In an adult, IGF-I circulates in serum at ∼ [42], [68]. Given that for each mature IGF-I protein there is one E-peptide produced, and alternative splicing under normal conditions generates 90% of the *Igf1* in the A form [25], serum EA would be approximately 45 nM while EB would only be at 5 nM. The underlying assumption is that the E-peptides enter the circulation similarly to IGF-I, and that they are stable, but given the short half-life of EB, it is unlikely to accumulate in tissues or the blood. Further, both predicted levels are below the sensitivity for detection by EA [31] or EB antibodies [69]. Thus, establishing that the E-peptides exist *in vivo* is difficult, at best. Our data support that the E-peptides work with IGF-I to modulate activity. The simplest way for this to occur is if they were still bound together as pro-IGF-I, which occurs *in vivo* [31], [32], [33]. Thus, while others have argued for independent actions of the E-peptides, we assert that a more plausible model is that E-peptide “activity” reflects actions of proIGF-I.

MGF is thought to activate satellite cells and increase proliferation at the expense of differentiation. The process by which MGF works has been deemed the “MGF hypothesis” (reviewed in [34], [59]). According to the hypothesis, there is preferential splicing to produce the *Igf1b* isoform immediately after muscle exercise or injury. Increased MGF/EB then activates satellite cells and promotes their proliferation. While the cells are proliferating, RNA processing reverts back to predominantly *Igf1a* isoforms, causing MGF/EB levels to decrease, and allowing differentiation to proceed and repair the injured muscle. Our results are, in part, consistent with this hypothesis, because we also observe that EB increases myoblast proliferation. If the return to *Igf1a* expression marks a switch between cell division and cell maturation, one would presume that EA would drive the next steps in muscle formation, namely differentiation and fusion, rather than continue to enhance proliferation. However, the inhibitory effects of EA on differentiation do not fit with this model. Also, in a recent study, specific targeting of either *Igf1a* or *Igf1b* delayed myoblast differentiation [52], suggesting that both E-peptides are necessary for normal differentiation. However, this would imply that increased EA or EB would enhance differentiation, contrary to our results. EB may possess much of the activity proposed in the MGF hypothesis, but EA shares similar activity.

Clearly, IGF-I and the E-peptides are not the sole determinants of the muscle formation process. Several additional growth factors are involved, and in some cases may be more efficient in driving these steps. For example, MGF/EB actions on myoblast migration have been attributed to the modulation of the matrix metalloproteinases (MMPs) [40], which are important regulators of muscle remodeling (reviewed in [70]). We, too, have found that EB enhances expression of MMP13, an interstitial collagenase important for wound healing [71], [72], [73]. Whether or not MMPs are one of the mechanisms involved in EB enhanced migration has not been addressed. However, since MEK inhibition did not completely block the migration response to EB, it suggests that other mechanisms coordinate with the MAPK pathway to mediate cell migration.

Could the E-peptides provide therapeutic benefit to muscle disease or damage? While IGF-I is widely recognized for its positive actions on muscle, modulatory factors such as the E-peptides may augment tissue responses to IGF-I. However, the interactions between the multiple products of the *Igf1* gene extend beyond muscle growth. For instance, targeting E-peptide activity could also prevent IGF-I mediated actions, which is a critical strategy for several anti-cancer therapies [74]. Regardless of the pro- or anti-growth intentions for IGF treatments, it is becoming clear that the E-peptides contribute to the actions of IGF-I, and should therefore be part of the equation for evaluation of IGF-I based therapies. Understanding the biological basis for E-peptide activity will help in clarifying IGF-I function.

## Materials and Methods

### Synthetic E-peptides

Murine EA and EB (based from GenBank AY878192 and AY878193, respectively) were synthesized by Bio-Synthesis Inc., Lewisville, TX, and purified via HPLC to >95%. The final products were confirmed via MALDI mass spectrometry (Wistar Proteomics Facility, U of Pennsylvania, Philadelphia, PA). EA and EB peptides begin at histidine 78, immediately following the SPC cleavage site [28]. The Scramble peptide sequence was created by randomly selecting 31 amino acids from EA and EB sequences (Figure 1B). Peptides were provided in 0.1 mg lyophilized aliquots to avoid freeze-thaw cycles and stored at −80°C until time of use.

### Synthetic E-peptide signaling

C2C12 (ATCC, Manassas, VA) cells were maintained in growth media (DMEM containing, 10% fetal bovine serum, and gentamicin). For signaling experiments, 2×10^4^ cells were seeded in 6-well plates in growth media and allowed to attach and grow overnight. The next day, cells were starved overnight in growth media without serum. On the third day, cells were treated with synthetic E-peptides and/or recombinant human IGF-I (Gemini Bio-Products West Sacramento, CA) for the time periods indicated in figures, and the cells were processed for immunoblotting as described below. For IGF-IR inhibition, 100 nM NVPAEW541 [47] diluted in DMSO or DMSO only was added to starved cells 90 minutes before and during treatment with synthetic peptides.

### Immunoblotting analysis

Signaling pathway activation was determined by immunoblotting. Cells were washed in cold PBS before incubation in lysis buffer (50 nM HEPES, 150 nM NaCl, 5 mM EDTA, 1 nM EGTA, 15 mM p-Nitrophenyl phosphate disodium hexahydrate, 1% NP-40, 0.1% SDS, 1% Deoxycholate, 0.025% Sodium Azide) with protease and phosphatase inhibitors (P8340, P5726, Sigma, St. Louis, MO). Debris were pelleted, and the total protein was measured in the supernatant. Equal amounts of protein were separated by SDS-PAGE and transferred to polyvinylidene fluoride membranes (Immobilon-P, Millipore, Bedford, MA). Membranes were blocked in Tris-buffered saline (TBS) plus 0.1% Tween 20 (TTBS) and 5% nonfat dry milk. Membranes were incubated in primary antibody diluted in 5% milk-TTBS overnight at 4°C. The following antibodies were used: phospho-Akt (no. 9271), phospho-ERK1/2 (no. 9101), total ERK1/2 (no. 9102), GFP (no. 2955) (Cell Signaling, Beverly, MA), and tubulin (T5168 Sigma). Membranes were washed in 5% milk-TTBS and incubated with horseradish peroxidase-conjugated secondary antibodies. Protein detection was performed using enhanced chemiluminescence and the ImageQuant (GE Fairfield, CT) detection system. Analysis of band intensity was performed using the associated image analysis software. Synthetic EA and EB stability was evaluated by incubating peptides in growth media at 37°C. Aliquots were obtained at 0–24 hours, and immunoblotted as above. An antibody to the EA peptide was generated by Bio-Synthesis Inc., Lewisville, TX and serum from the inoculated rabbit was used to visualize EA. An antibody to MGF/EB was used to visualize the EB peptide [69].

### IGF-IR activation and location assays

To determine if E-peptides directly activate IGF-IR, a KIRA assay was preformed as previously described [75] with a few alterations. Briefly, 2.5×10^4^ P6 cells, which overexpress IGF-IR (another kind gift from the Baserga lab [49]) were seeded into 96-well plates. They were maintained in growth media supplemented with 200 µg/ml G418. The cells were starved for 6 hours, and then treated with synthetic E-peptides and/or IGF-I for 15 minutes. Cells were lysed and IGF-IR was captured onto an ELISA plate coated with an antibody to IGF-IR (MAB1120, Millipore, Billerica, MA). An HRP-conjugated antibody to phosphorylated tyrosines (16–454, Millipore) and TMB substrate (N301, Thermo Scientific, Rockfort, IL) was used for colorimetric quantification. Absorbance was read at 450 nm via the SpectraMax M5 plate reader (Molecular Devices, Sunnyvale, CA), which served as an indicator of IGF-IR phosphorylation. The IGF-IR localization assay was based on [50], [75], [76] and preformed as described above, except after treatment and before lysis, cell surface proteins were labeled with 0.3 mg/ml sulfo-NHS-biotin (21217, Thermo Scientific, Rockfort, IL) in PBS for 1 hour at 4°C. Two ELISA plates coated as above were utilized. Half of the cell lysates were transferred to one plate to measure cell-surface IGF-IR, visualized by an HRP-conjugated strepavidin antibody. The remaining lysates were used on the second plate to measure total IGF-IR, by incubation with an IGF-IR antibody (C-20, Santa Cruz, CA) followed by an a HRP-conjugated anti-rabbit antibody.

### Cell proliferation

Proliferation was measured using a 5-bromo-2P-deoxyuridine (BrdU) plate and slide assays (Roche, Indianapolis, IN). For the plate assay, 5×10^3^ C2C12 cells were seeded in 96-well plates. Cells were starved for 6 hours, and treated with synthetic E-peptides or recombinant IGF-I overnight. BrdU was added for incorporation for 2 hours before cell lysis and BrdU staining. For MAPK inhibition, a MEK inhibitor was added to the media (PD 098059, Sigma, 50 µM). For IGF-IR inhibition, 100 nM NVPAEW541 was added to the media. The slide assay was done as above, except with 2×10^4^ C2C12 cells in 24-well plates on glass cover slips. Cells were stained with an antibody to BrdU and with DAPI (VectorLabs, Burlingame, CA) to visualize nuclei. Images were acquired using a Leica DMR epifluorescence microscope using OpenLab imaging software (Improvision, PerkinElmer, Waltham, MA).

### Cell migration

E-peptide effects on cell migration were tested using a 24-well Transwell (8.0 µm pore size) plate assay (Corning Inc., Lowell, MA). C2C12 cells (2×10^4^) were seeded in the upper chambers in serum-free media, and synthetic E-peptides in serum-free media were placed in the bottom chambers. Cells were allowed to migrate for 5 hours. Non-migrated cells remaining in the upper chamber were removed from the transwell membranes with Q-tips and migrated cells were fixed with 4% formaldehyde and stained with DAPI. The transwell membranes were mounted onto slides, and imaged at 10× using above-mentioned microscopy and analysis software. For MAPK signaling or IGF-IR inhibition, PD (50 µM) or NVP (100 nM) was added to both chambers at the start of the experiment.

### Muscle cell differentiation

Cells were plated in 6-well plates and changed to differentiation media (DMEM containing, 2% horse serum, and gentamicin) when they reached 80–90% confluency (Day 0). Differentiation media was changed every day until Day 3. Synthetic E-peptides (100 nM) were added to the differentiation media once a day. Total RNA was isolated from differentiating cells using Trizol (Invitrogen, Carlsbad, CA).

### Quantitative RT-PCR

Equal amounts of total RNA from each sample were subjected to single-strand reverse transcription (Applied Biosystems, Foster City, CA). The resultant cDNA was utilized for quantitative RT-PCR (qRT-PCR) with oligonucleotides specific for genes listed below using the Applied Biosystems 7300 Real-Time PCR System, and reagents (Power SYBR Green PCR Master Mix). All samples were loaded in duplicate in 96-well plates. Expression of 18S was used to control for cDNA content. Fold change was calculated by comparing ΔCT values for each gene at each Day to ΔCT at Day 0. Primers used: 18S, 5′-ctctgttccgcctagtcctg-3′ and 5′-aatgagccattcgcagtttc-3′; MyoD, 5′-tgctcctttgagacagcaga-3′ and 5′-agtagggaagtgtgcgtgct-3′; Myogenin, 5′-gggcccctggaagaaaag-3′ and 5′-aggaggcgctgtgggagt-3′; Embryonic myosin, 5′-gcatagctgcacctttcctc-3′ and 5′-cgtgtatcggtccttgaggt-3′; IGF-I, 5′-cacacctcttctacctggcgctctgc-3′ and 5′-agtctcctcagatcacagctccg-3′.

### Statistical analysis

All data was analyzed via student t-tests, 1-way ANOVA followed by a Tukey post-test, or by 2-way ANOVA followed by a Bonferroni post-test. Statistical significance was accepted at p<0.05.
